# Survival and Complication of Liver Transplantation in Infants: A Systematic Review and Meta-Analysis

**DOI:** 10.3389/fped.2021.628771

**Published:** 2021-04-29

**Authors:** Yifu Hou, Xiaoxiao Wang, Hongji Yang, Shan Zhong

**Affiliations:** ^1^Department of Organ Transplantation, Sichuan Provincial People's Hospital, University of Electronic Science and Technology of China, Chengdu, China; ^2^Chinese Academy of Sciences Sichuan Translational Medicine Research Hospital, Chengdu, China

**Keywords:** liver transplant, infants, meta-analysis, survival, complication

## Abstract

**Background:** Modern surgical techniques and scientific advancements have made liver transplant (LT) in infants feasible. However, there are only a small number of studies examining the short- as well as long-term outcomes of LT in this vulnerable subset of children.

**Methods:** Comprehensive searches were done systematically through the PubMed, Scopus, and Google scholar databases. Studies that were retrospective record based or adopted a cohort approach and reported either patient survival rates or graft survival rates or complications of LT in infants were included in the meta-analysis. Statistical analysis was done using STATA version 13.0.

**Results:** A total of 22 studies were included in the meta-analysis. The overall pooled patient survival rate at 1 year, >1–5 years, and >5 years post-transplantation was 85% (95% CI: 78-−92%), 71% (95% CI: 59–83%), and 80% (95% CI: 69–91%), respectively. The overall pooled graft survival rate at 1 year, >1–5 years, and >5 years post-transplantation was 72% (95% CI: 68–76%), 62% (95% CI: 46–78%), and 71% (95% CI: 56–86%), respectively. The overall pooled rate for vascular complications, need for re-transplantation, biliary complications, and infection/sepsis was 12% (95% CI: 10–15%), 16% (95% CI: 12–20%), 15% (95% CI: 9–21%), and 50% (95% CI: 38–61%), respectively.

**Conclusion:** The current meta-analysis showed modest patient and graft survival rates for infant liver transplantation. However, the complication rates related to infection/sepsis were high. More comprehensive evidence is required from studies with larger sample sizes and a longer duration of follow-up.

## Introduction

Modern surgical techniques and scientific advancements have made liver transplant (LT) in infants feasible. During the last two decades, for infants (i.e., children with age <12 months) with end-stage liver disease, LT has emerged as a life-saving medical procedure (1, 2). It is important to understand that the indications for LT between infants and older children vary and so is the acuity and severity of the consequent liver disease. Conditions such as hemochromatosis, certain metabolic disorders, viral hepatitis including hepatitis B, enteroviral and echoviral hepatitis, and idiopathic giant cell hepatitis are common indications in infants, whereas diseases causing chronic cholestasis are more commonly seen with older children (3, 4). Studies have documented that of all pediatric candidates, infants have had the highest rates of wait-list mortality (5, 6). However, in this youngest and most vulnerable group of children, there are only a small number of studies examining the outcomes of LT. Critical care advances and novel immunosuppressive agents have dramatically altered the management system of children needing liver transplantation. As a result, the outcomes of these children, especially young children, have improved considerably. Most of the available studies documenting outcomes of LT in infants are limited to single-center and the reason is largely due to the rarity of LT in this age group (7, 8). Available data suggest that around 2% of the total liver transplants in the pediatric age group are done in infants (9).

To the best of our knowledge, no study has attempted to synthesize evidence on the survival and complications with LT in infants. Aggregated evidence from multiple studies would help to provide better data for decision making and treatment planning in this vulnerable group of children. Therefore, the aim of this study is to systematically search literature and perform a meta-analysis evaluating patient and graft survival as well as complications in infants undergoing LT.

## Methods

### Search Strategy

A comprehensive search was done systematically through the PubMed, Scopus, and Google scholar databases for English as well as non-English language papers published up to 1st October 2020. For non-English language papers, the Google translator was used for translation to English and, thereafter, extract relevant information. Free text words and medical subject heading (MeSH) terms were used. Details of the search strategy have been provided in a supplementary document (Supplementary Table 1).

### Selection Criteria and Methods

Two authors reviewed the citations and selected studies. After removing the duplicates, screening of titles and abstracts was performed as a first step. Thereafter, review of the full text of potential studies was done. Any discrepancies related to the inclusion of studies were resolved through detailed discussion among the study authors. Only those studies that adequately suited the inclusion criteria were selected for the meta-analysis. The bibliographic list of the identified studies and relevant reviews on the subject were examined for additional possible studies.

#### Inclusion Criteria

For a study to be included in the meta-analysis, it should have reported either patient survival rates or graft survival rates or complications of LT in infants. Studies that were retrospective record based or adopted a cohort approach with follow-up of infants that received LT were included in the meta-analysis.

#### Exclusion Criteria

Case reports or review articles were excluded. Also, those studies that did not report on any outcomes of interest were excluded.

### Data Extraction and Quality Assessment

Extraction of relevant data from the included studies was done by two authors independently using a data extraction sheet. The following data from the eligible studies were extracted: the surname of the first author, the year in which the study was published, the geographical location where the study was done, the sample size, the design of the study, and the key findings of the study. The Newcastle-Ottawa Quality Assessment Scale adapted for observational studies was used for quality assessment of included studies (10).

### Statistical Analysis

Statistical analysis was done using STATA version 13.0. A meta-analysis of the reported prevalence in the included studies was done. The outcomes considered were patient survival rates, graft survival rates, and complication rates. For the patient and graft survival outcomes, the reported survival rates were analyzed as follows: (1) survival rate at 1 or within 1 year of transplant; (2) survival rate for the period between >1 and ≤ 5 years post-transplantation; and (3) survival rate at >5 years post-transplantation. All estimates were reported with a 95% confidence interval (CI). The final estimates of prevalence were reported as percentages with 95% CI. The heterogeneity of effects was assessed and quantified by the *I*^2^. *I*^2^ value >50% was considered to represent substantial heterogeneity (11). In cases with substantial heterogeneity, a random effects model was used. Subgroup analysis was done based on the timing of the study publication, i.e., those published before year 2000 and those from the year 2000 onwards. This was done to ascertain whether there were any differences in patient survival, graft survival, or complication rate before and after the year 2000, owing to technical and scientific advancements. The difference in the pooled proportions between the two subgroups was evaluated for their statistical significance. The subgroup analysis also aided in understanding whether the timing of conduct of the study had any influence on the heterogeneity of the effects obtained. For the two important outcomes in this review, i.e., the patient and the graft survival, we further conducted subgroup analysis based on the indication for transplant, type of donor (living or cadaveric), type of transplant (whole or reduced/split), and age and weight at the time of receiving transplant. A *p* < 0.05 was considered statistically significant. Publication bias was assessed using Begg's test and visually inspected using funnel plots.

## Results

### Selection of Articles, Study Characteristics, and Quality of Included Studies

**Figure 1:**
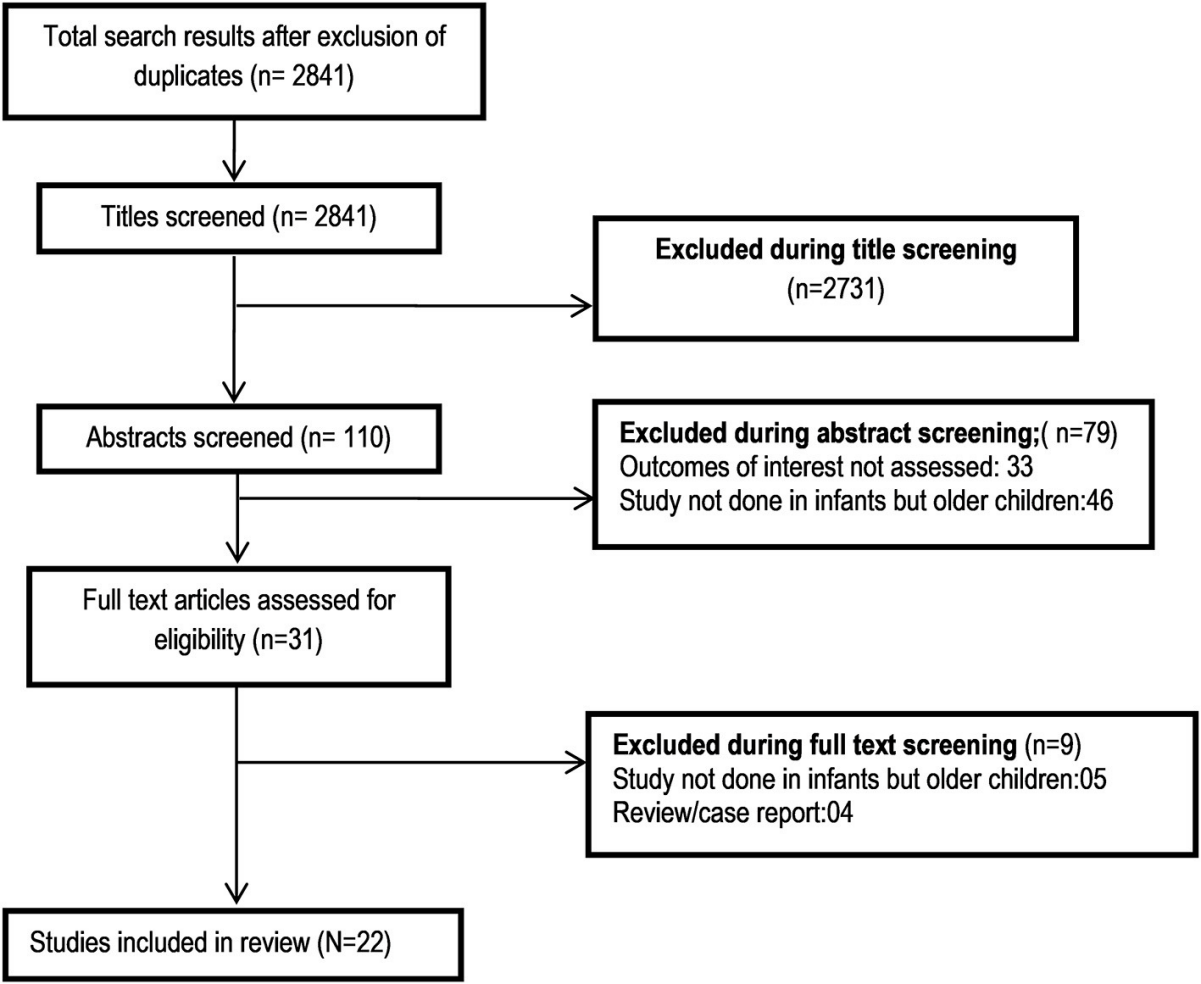
Selection process of the studies included in the review.

A total of 2,841 unique citations were obtained upon executing the search strategy in the PubMed, Scopus, and Google scholar databases (Figure 1). Out of these, 2,731 were excluded based on title screening. Furthermore, 79 citations were excluded after reading of the abstract. The full text of the remaining 31 articles was reviewed. Out of these, nine articles were excluded upon the full text review. The final number of included articles in this meta-analysis was 22 (12–33). Supplementary Table 3 presents the key characteristics of the included studies along with the key findings. Out of all the included studies, 10 studies were published before the year 2000, and the remaining 12 were published at or after the year 2000. Majority of the studies were done in the United States (12/22) followed by the United Kingdom (4/22). One study each was done in Canada, Belgium, Germany, Hong Kong, Japan, and Italy (Supplementary Table 3). All the included studies were non-randomized, and most were retrospective medical record based. Supplementary Table 2 presents the findings of the quality assessment of included studies. All the included studies had moderate to high quality (Supplementary Table 2).

### Pooled Evidence for Patient Survival

**Figure 2:**
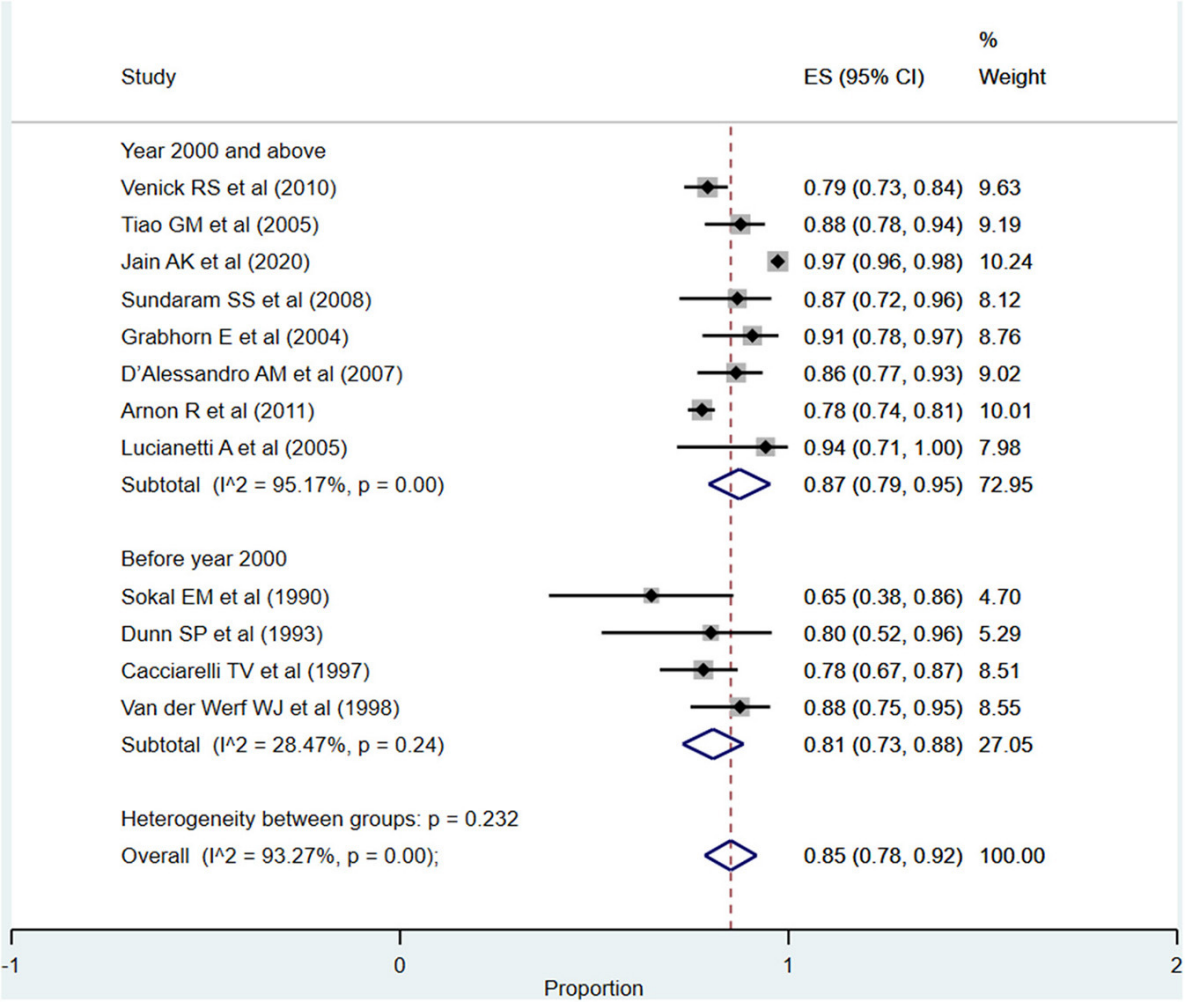
Pooled patient survival rate among infants at or within 1 year post-liver transplantation.

The overall pooled patient survival rate among infants at 1 year post-transplantation period was 85% (95% CI: 78–92%; *I*^2^ = 93.27%; Figure 2). There was no evidence of publication bias (Begg's *P* = 0.42; Supplementary Figure 1). When only studies published at or after the year 2000 were considered, the pooled survival rate was 87% (95% CI: 79–95%; *I*^2^ = 95.17%). The survival rate was lower when studies published before the year 2000 were pooled, i.e., 81% (95% CI: 73–88%; *I*^2^ = 28.47%). This observed difference in the pooled patient survival rate was statistically significant (*P* = 0.04).

**Figure 3:**
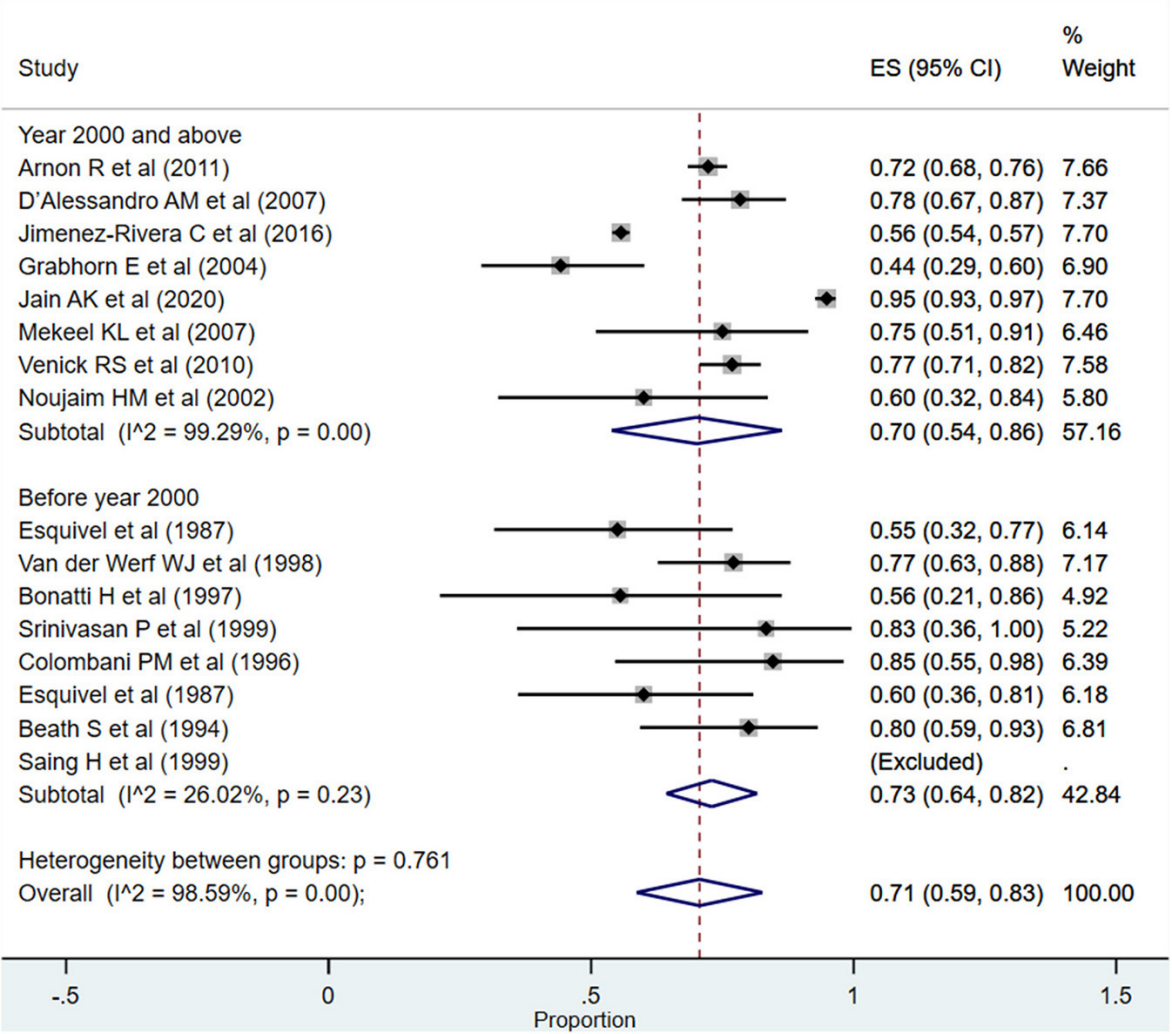
Pooled patient survival rate among infants between >1 and 5 years post-liver transplantation.

For the period between >1 and 5 years, the overall pooled survival rate was 71% (95% CI: 59–83%; *I*^2^ = 98.59%; Figure 3). Upon pooling of studies published at or after the year 2000, the pooled survival rate was 70% (95% CI: 54–86%; *I*^2^ = 99.29%). The pooled survival rate was 73% (95% CI: 64–82%; *I*^2^ = 26.02%) when studies published before the year 2000 were pooled. The observed difference in the survival rate was statistically not significant (*P* = 0.43).

**Figure 4:**
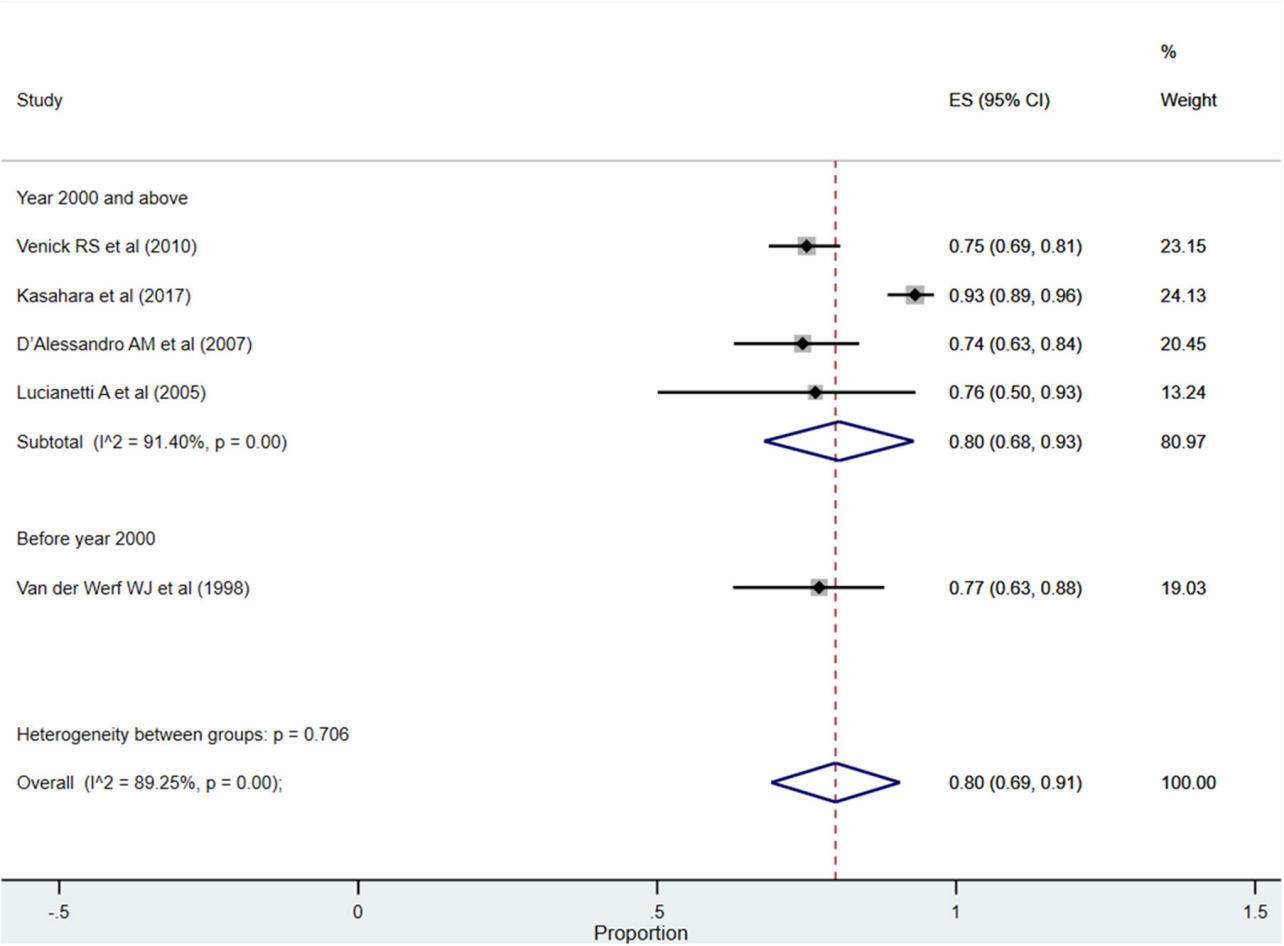
Pooled patient survival rate among infants at more than 5 years post-liver transplantation.

After more than 5 years post-transplantation, the overall pooled patient survival rate was 80% (95% CI: 69–91%; *I*^2^ = 89.25%; Figure 4). Upon pooling of studies published at or after the year 2000, the pooled survival rate was 80% (95% CI: 68–93%; *I*^2^ = 91.40%). The pooled survival rate was 77% (95% CI: 63–88%) when studies published before the year 2000 were pooled. The observed difference in the survival rate was statistically not significant (*P* = 0.62).

Even though the number of studies was small, the subgroup analysis, for the period between >1 and 5 years post-operatively, suggested an improved patient survival when the indication for transplant was biliary atresia/cholestasis [71%; 95% CI: 58–85%] compared to when the indication was acute hepatic failure/metabolic disorder [66%; 95% CI: 50–82%] (Table 1). Similarly, the survival was better when the graft was received from a living donor [77%; 95% CI: 60–94%], the type of graft was reduced/split [73%; 95% CI: 57–89%], the weight at the time of transplant was ≥6 kg [74%; 95% CI: 57–91%], and the age at the time of transplant was ≥6 months [74%; 95% CI: 56–92%] (Table 1).

**Table 1:** Findings of the subgroup analysis for patient and graft survival.

	***N*** **(no. of studies); pooled effect size (proportion) with 95% CI**									
	**Reason for transplant**		**Donor**		**Type of graft**		**Mean weight at time of transplant**		**Age at transplant**	
	**Biliary atresia/cholestatic disease**	**Acute hepatic failure/metabolic disorder**	**Living**	**Cadaveric**	**Whole**	**Reduced/split**	** <6 kg**	**≥6 kg**	** <6 months**	**≥6 months**
**PATIENT SURVIVAL**										
≤ 1 year	*N* = 11 0.85 (0.78, 0.92)	*N* = 1 0.87 (0.72, 0.96)	*N* = 1 0.65 (0.38, 0.86)	*N* = 11 0.86 (0.79, 0.93)	*N* = 5 0.81 (0.77, 0.85)	*N* = 7 0.88 (0.81, 0.95)	*N* = 4 0.87 (0.78, 0.95)	*N* = 8 0.84 (0.76, 0.92)	*N* = 5 0.87 (0.79, 0.94)	*N* = 7 0.84 (0.75, 0.93)
>1–5 years	*N* = 12 0.71 (0.58, 0.85)	*N* = 3 0.66 (0.50, 0.82)	*N* = 2 0.77 (0.60, 0.94)	*N* = 13 0.70 (0.58, 0.83)	*N* = 7 0.69 (0.59, 0.79)	*N* = 8 0.73 (0.57, 0.89)	*N* = 6 0.65 (0.53, 0.77)	*N* = 9 0.74 (0.57, 0.91)	*N* = 7 0.67 (0.58, 0.77)	*N* = 8 0.74 (0.56, 0.92)
>5 to 10 years	*N* = 5 0.80 (0.69, 0.91)	–	*N* = 1 0.93 (0.89, 0.96)	*N* = 4 0.75 (0.71, 0.80)	*N* = 3 0.75 (0.71, 0.80)	*N* = 2 0.76 (0.50, 0.93)	*N* = 1 0.76 (0.50, 0.93)	*N* = 4 0.80 (0.69, 0.92)	*N* = 2 0.77 (0.67, 0.87)	*N* = 3 0.81 (0.67, 0.95)
**GRAFT SURVIVAL**										
≤ 1 year	*N* = 12 0.72 (0.67, 0.76)	*N* = 1 0.76 (0.60, 0.89)	*N* = 1 0.65 (0.37, 0.87)	*N* = 12 0.72 (0.68, 0.77)	*N* = 7 0.68 (0.65, 0.71)	*N* = 6 0.79 (0.72, 0.85)	*N* = 4 0.78 (0.66, 0.91)	*N* = 9 0.70 (0.65, 0.75)	*N* = 5 0.76 (0.66, 0.86)	*N* = 8 0.70 (0.65, 0.75)
>1–5 year	*N* = 10 0.63 (0.45, 0.81)	*N* = 3 0.57 (0.39, 0.75)	*N* = 2 0.69 (0.48, 0.89)	*N* = 11 0.61 (0.44, 0.79)	*N* = 5 0.61 (0.47, 0.76)	*N* = 8 0.63 (0.42, 0.83)	*N* = 6 0.67 (0.55, 0.64)	*N* = 7 0.67 (0.42, 0.91)	*N* = 7 0.60 (0.55, 0.65)	*N* = 6 0.66 (0.40, 0.93)
>5–10 year	*N* = 5 0.71 (0.56, 0.86)	–	*N* = 1 0.90 (0.85, 0.94)	*N* = 4 0.65 (0.58, 0.72)	*N* = 3 0.64 (0.56, 0.71)	*N* = 2 0.90 (0.86, 0.94)	*N* = 1 0.76 (0.50, 0.93)	*N* = 4 0.70 (0.53, 0.87)	*N* = 2 0.68 (0.57, 0.80)	*N* = 3 0.72 (0.52, 0.92)

### Pooled Evidence for Graft Survival

**Figure 5:**
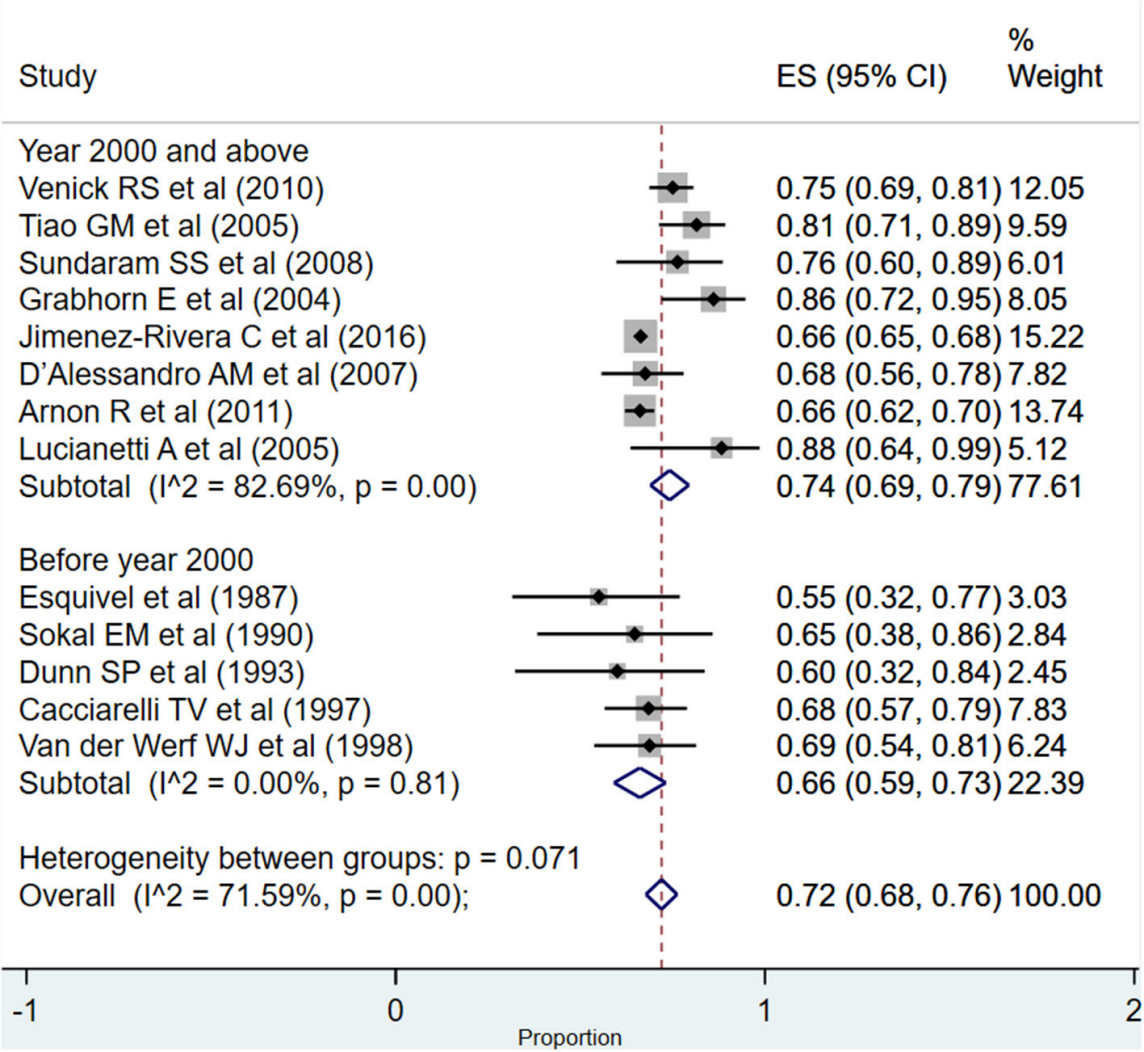
Pooled graft survival rate among infants at or within 1 year post-liver transplantation.

The overall pooled graft survival rate at 1 year post-transplantation was 72% (95% CI: 68–76%; *I*^2^ = 71.59%; Figure 5). There was no evidence of publication bias (Begg's *P* = 0.46; Supplementary Figure 2). The pooled survival rate was 74% (95% CI: 69–79%; *I*^2^ = 82.69%) when studies published at or after the year 2000 were considered. The survival rate was comparatively lower when studies published before the year 2000 were pooled, i.e., 66% (95% CI: 59–73%; *I*^2^ = 0.00%), and this difference in the pooled graft survival rate was statistically significant (*P* = 0.02).

**Figure 6:**
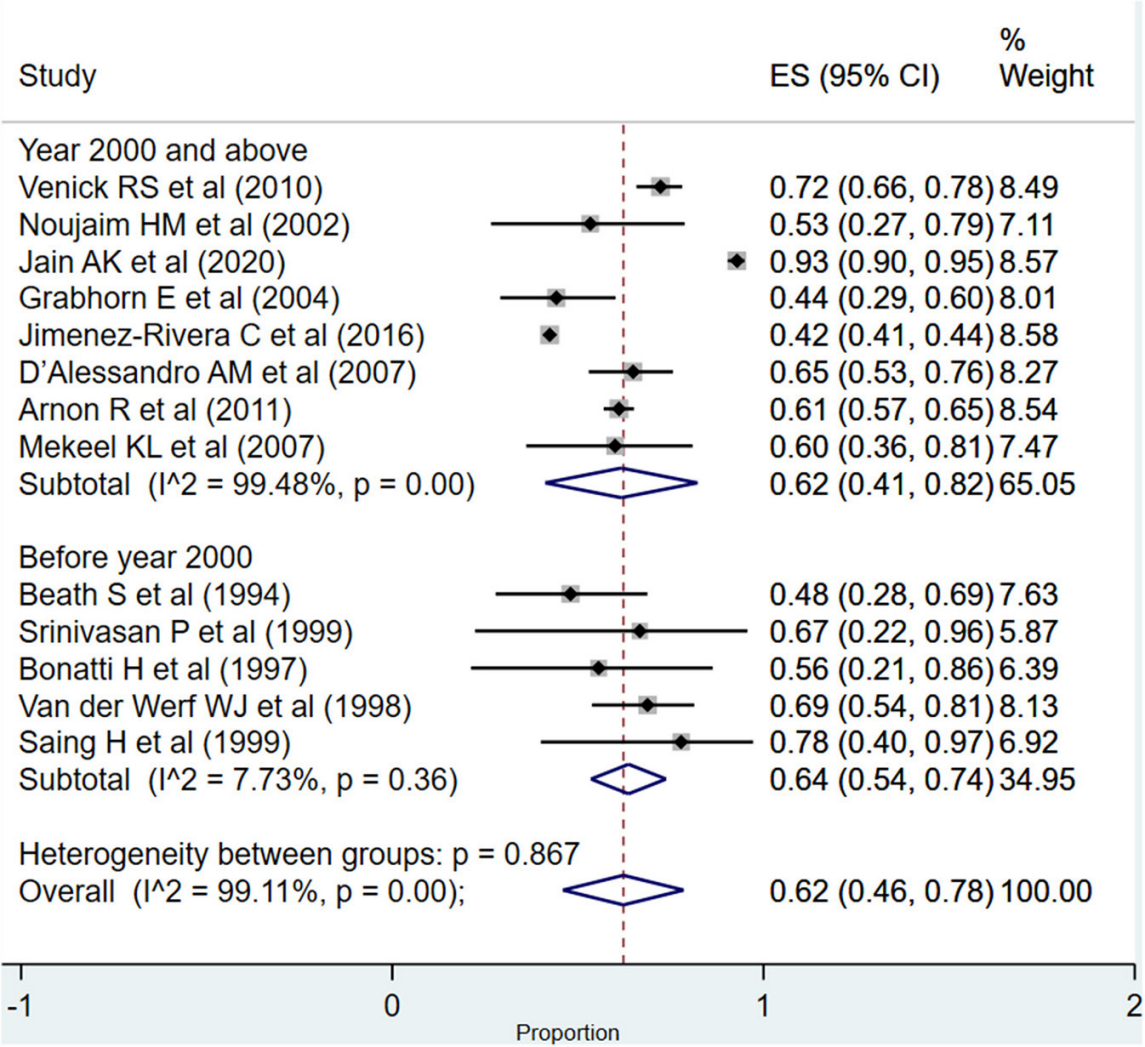
Pooled graft survival rate among infants between >1 and 5 years post-liver transplantation.

For the period between >1 and 5 years, the overall pooled survival rate was 62% (95% CI: 46–78%; *I*^2^ = 99.11%; Figure 6). In the subgroup analysis, based on the time of publication of the included studies, the pooled graft survival rates across the two subgroups, i.e., studies published at or after the year 2000 (62%; 95% CI: 41–82%; *I*^2^ = 99.48%) and those published before the year 2000 (64%; 95% CI: 54–74%; *I*^2^ = 7.73%), were statistically similar (*P* = 0.68).

**Figure 7:**
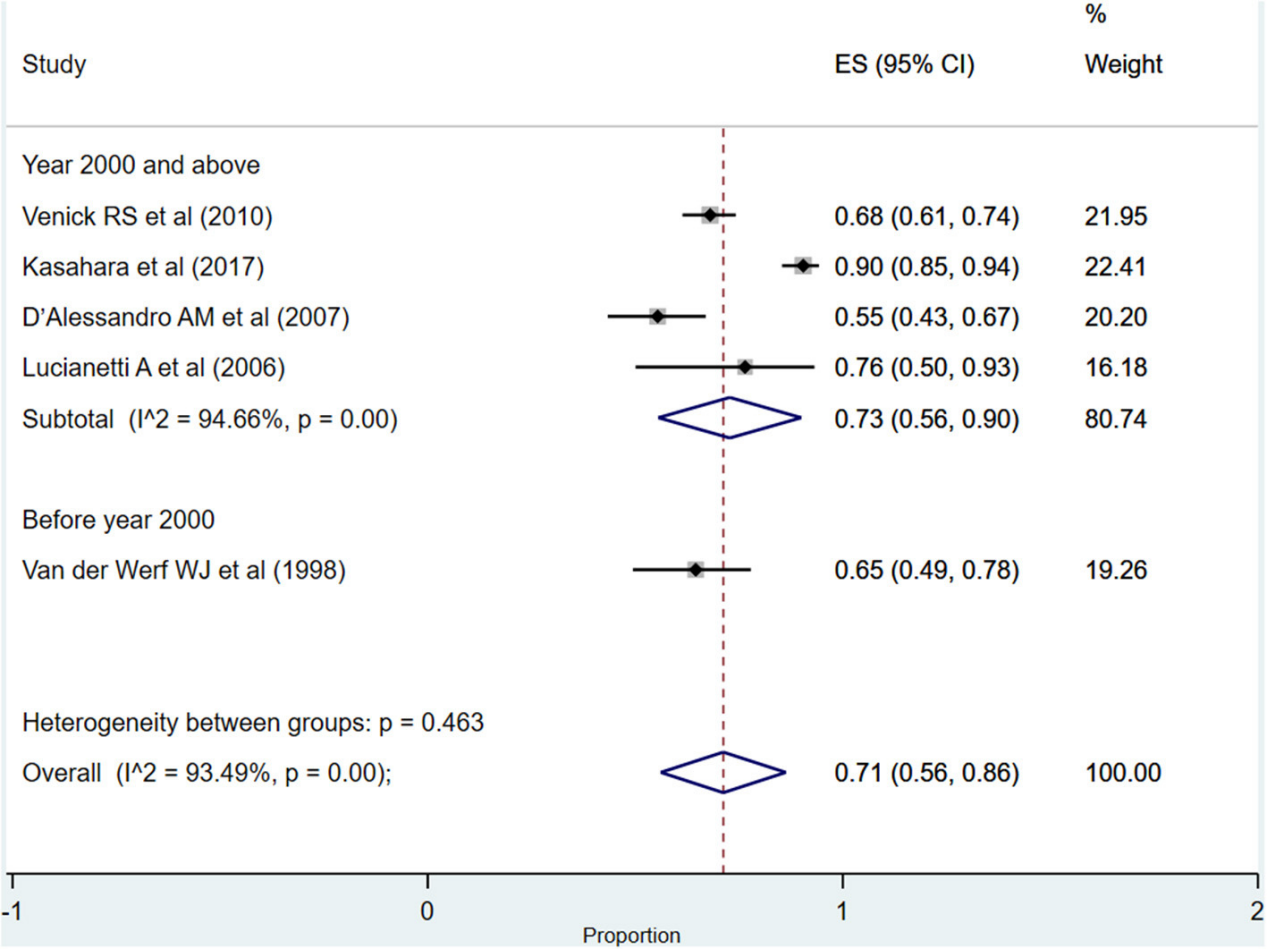
Pooled graft survival rate among infants at more than 5 years post-liver transplantation.

After more than 5 years post-transplantation, the overall pooled graft survival rate was 71% (95% CI: 56–86%; *I*^2^ = 93.49%; Figure 7). Upon pooling of studies published at or after the year 2000, the pooled survival rate was 73% (95% CI: 56–90%; *I*^2^ = 94.66%). The pooled survival rate was 65% (95% CI: 49–78%) when studies published before the year 2000 were pooled. The observed difference in the survival rate was statistically not significant (*P* = 0.23).

The subgroup analysis, for the period between >1 and 5 years post-operatively, suggested an improved graft survival when the indication was biliary atresia/cholestasis [63%; 95% CI: 45–81%] compared to acute hepatic failure/metabolic disorder [57%; 95% CI: 39–75%] (Table 1). The graft survival was better when it was received from a living donor [69%; 95% CI: 48–89%] and the age at the time of transplant was ≥6 months [66%; 95% CI: 40–93%] (Table 1). Further details of the subgroup analysis have been provided in Table 1.

### Complications Post-transplantation

#### Vascular Complications (Include Hepatic Artery Thrombosis and Portal Vein Thrombosis)

**Figure 8:**
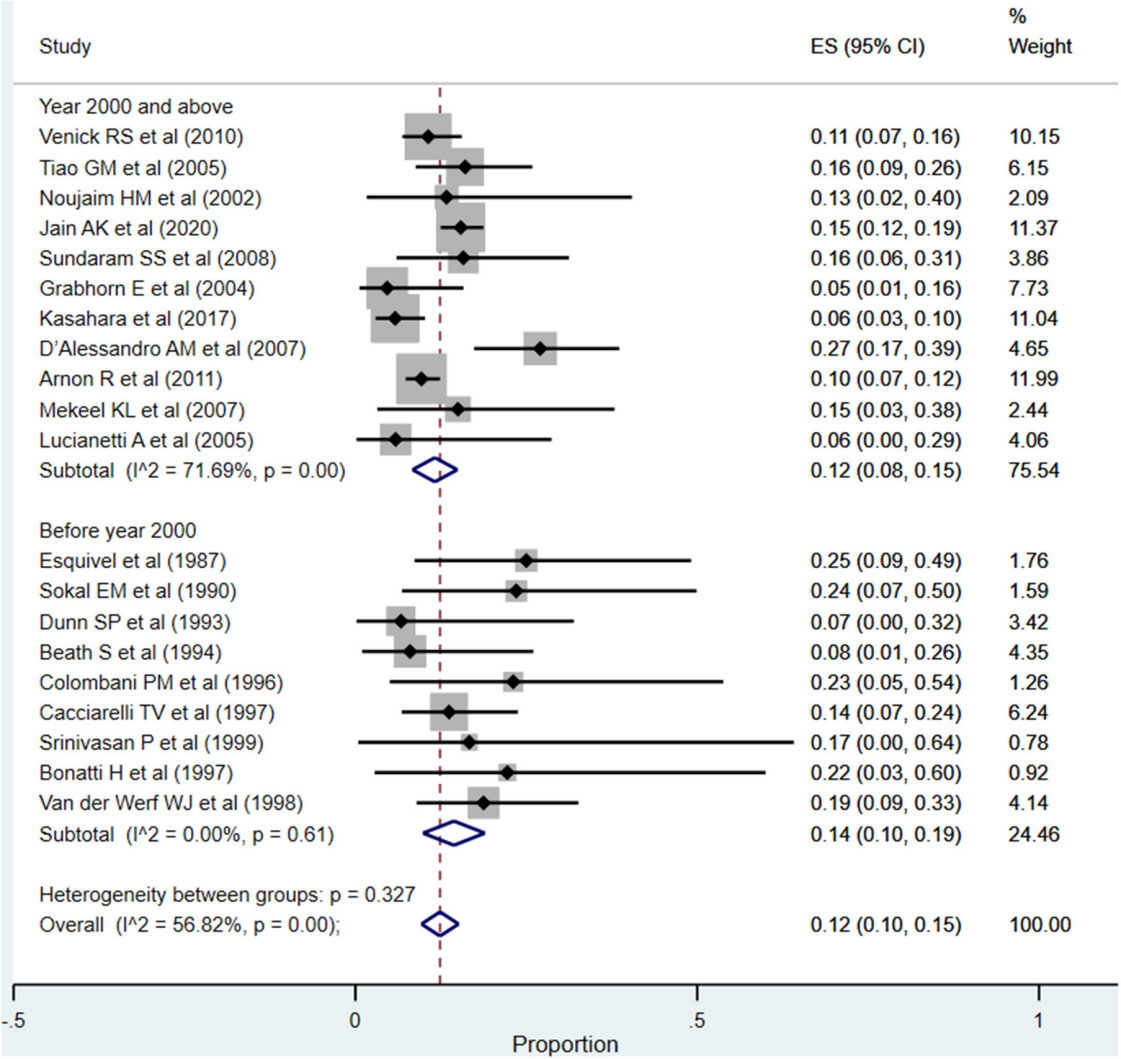
Pooled rate for vascular complications (hepatic artery thrombosis and portal vein thrombosis) in the included studies.

The overall pooled rate for vascular complications was 12% (95% CI: 10–15%; *I*^2^ = 56.82%; Figure 8). In the subgroup analysis, based on the time of publication of the included studies, the pooled vascular complication rates across the two subgroups, i.e., studies published at or after the year 2000 (12%; 95% CI: 8–15%; *I*^2^ = 71.69%) and those published before the year 2000 (14%; 95% CI: 10–19%; *I*^2^ = 0.00%), were statistically similar (*P* = 0.38).

#### Re-transplantation

**Figure 9:**
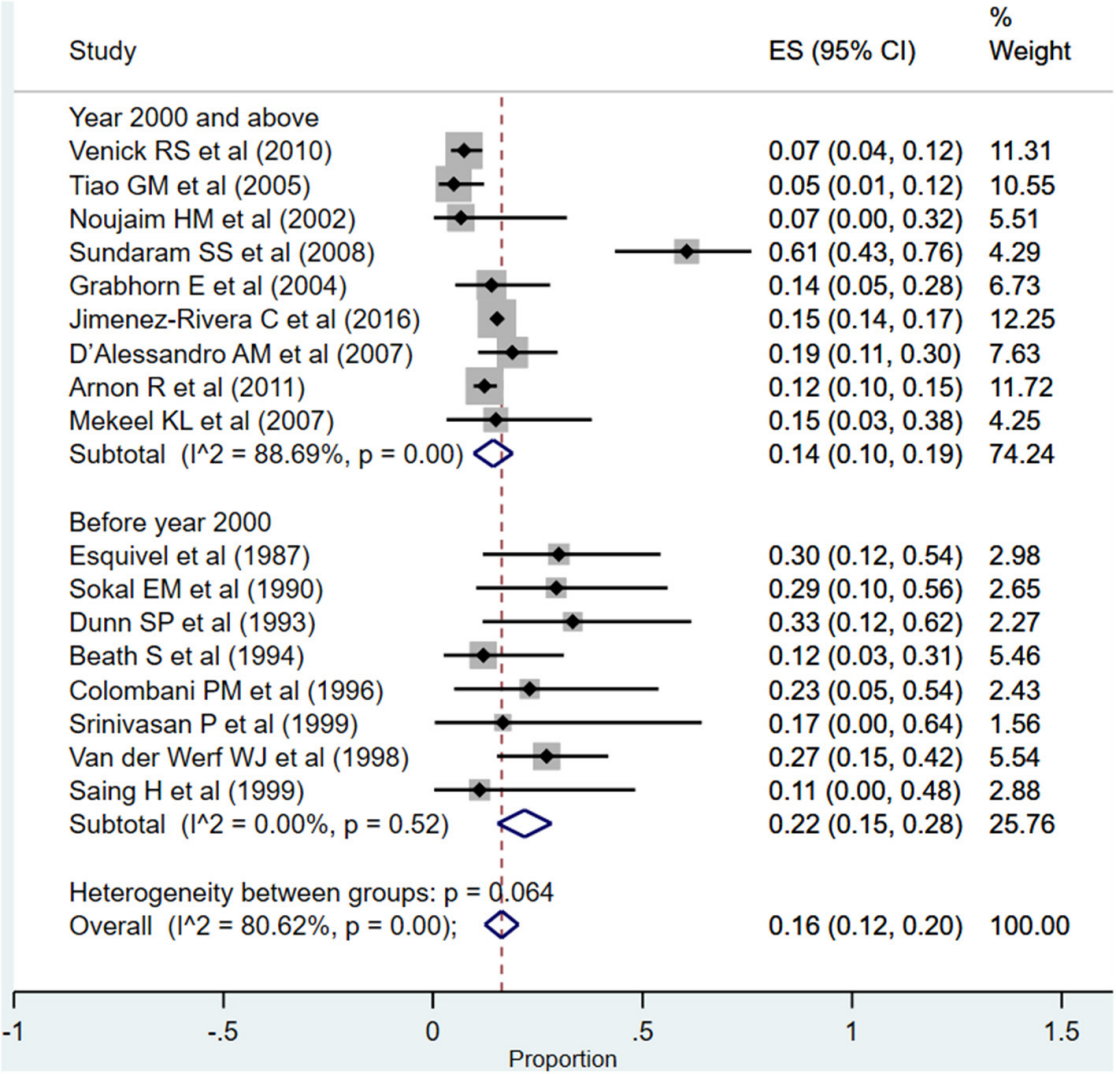
Pooled rate for re-transplantation in the included studies.

The overall pooled rates for need for re-transplantation was 16% (95% CI: 12–20%; *I*^2^ = 80.62%; Figure 9). The rate was significantly higher when studies published before the year 2000 were pooled (22%; 95% CI: 15–28%; *I*^2^ = 0.00%) compared to when studies published at or after the year 2000 were pooled (14%; 95% CI: 10–19%; *I*^2^ = 88.69%) (*P* = 0.005).

#### Biliary Complications

**Figure 10:**
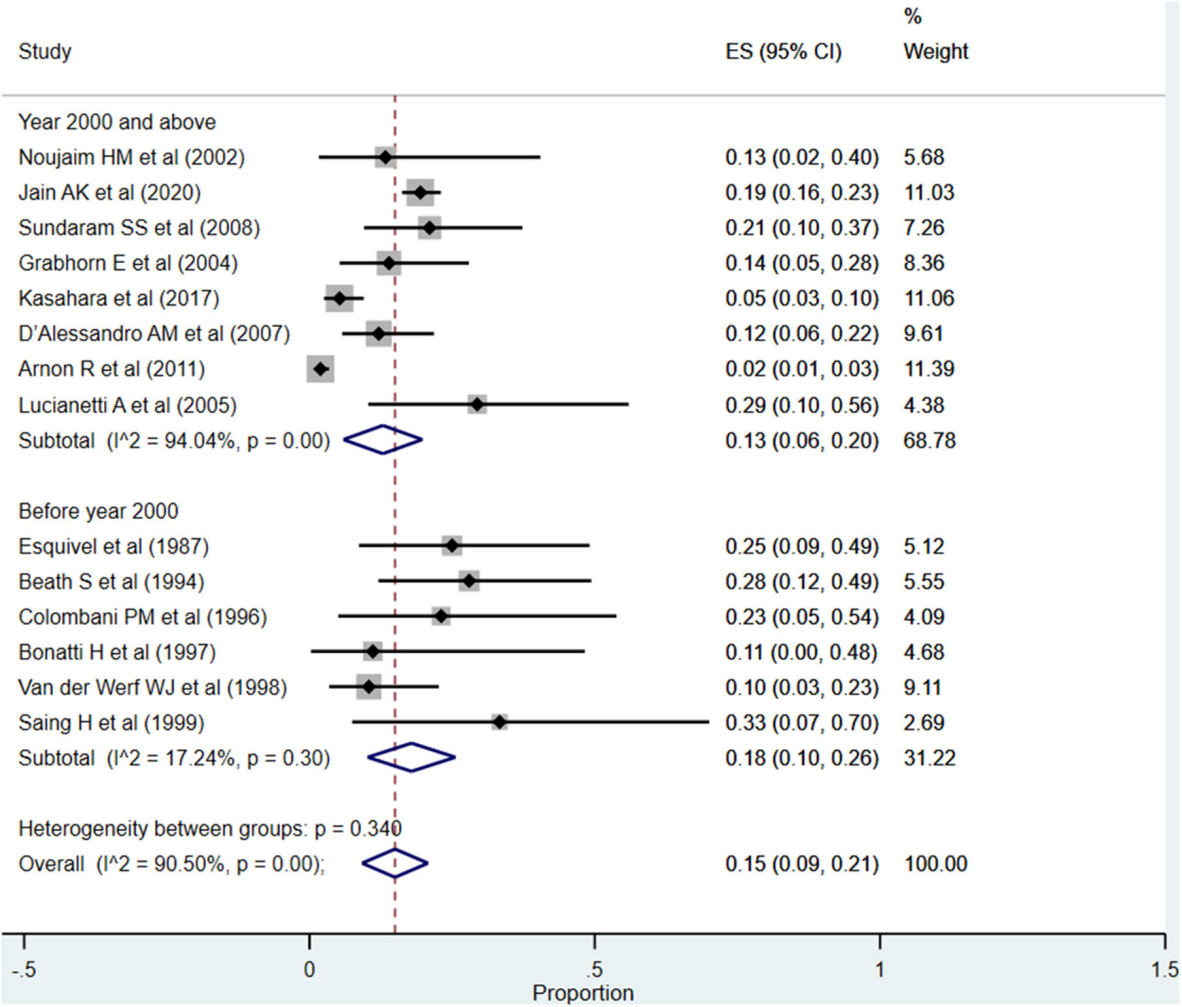
Pooled rate for biliary complications in the included studies.

The overall pooled rate for biliary complications was 15% (95% CI: 9–21%; *I*^2^ = 90.50%; Figure 10). Although the rate was significantly higher when studies published before the year 2000 were pooled (18%; 95% CI: 10–26%; *I*^2^ = 17.24%), compared to when studies published at or after the year 2000 were pooled (13%; 95% CI: 6–20%; *I*^2^ = 94.04%), this difference did not reach statistical significance (*P* = 0.11).

#### Infections/Sepsis

**Figure 11:**
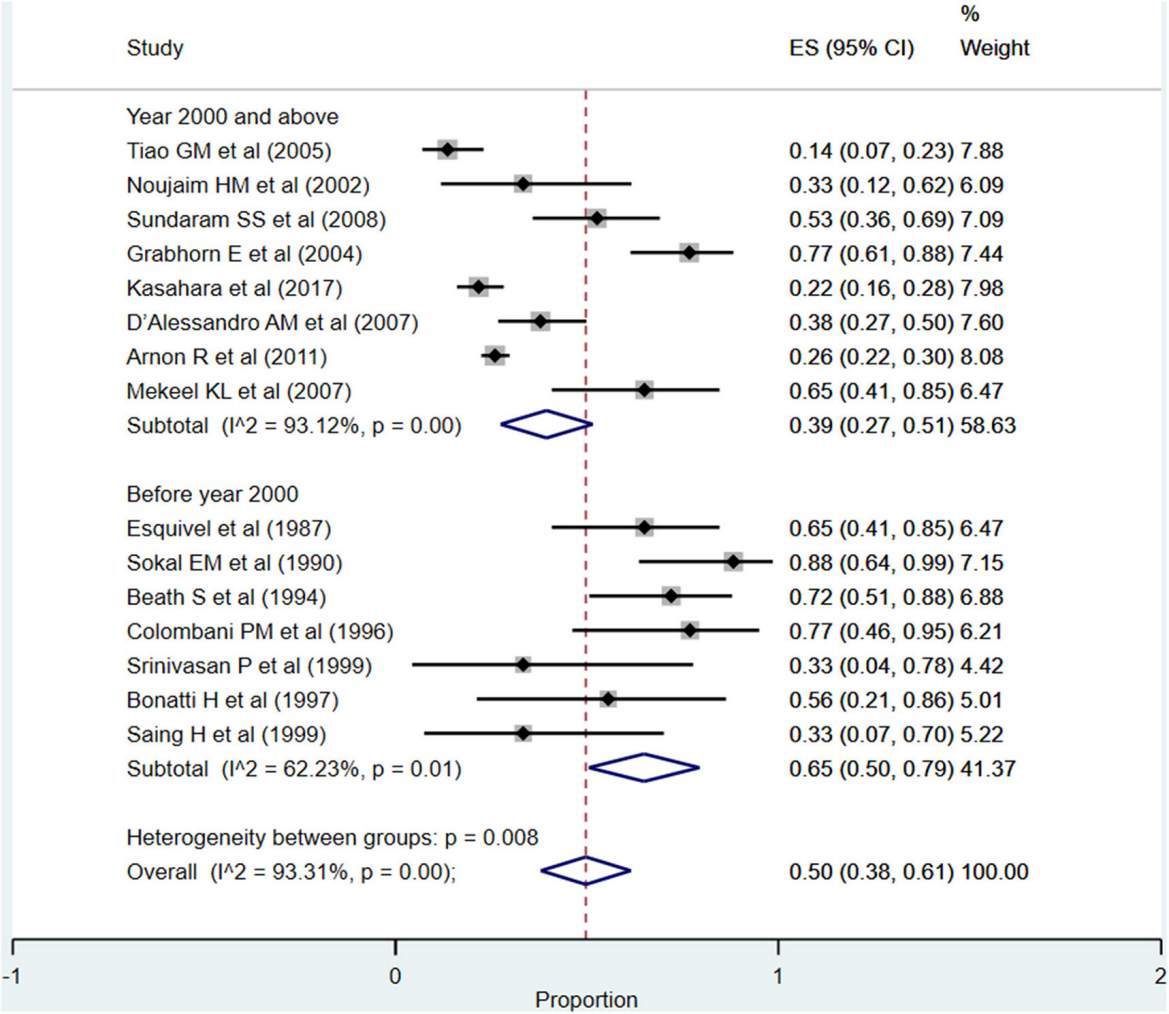
Pooled proportion for infections/sepsis in the included studies.

The overall pooled rate for infections and/or sepsis following liver transplantation was high, i.e., 50% (95% CI: 38–61%; *I*^2^ = 93.31%; Figure 11). A comparatively higher rate was noted when studies published before the year 2000 were pooled (65%; 95% CI: 50–79%; *I*^2^ = 62.23%) compared to when studies published at or after the year 2000 were pooled (39%; 95% CI: 27–51%; *I*^2^ = 93.12%) (*P* = 0.001).

## Discussion

Liver transplantation is often the only cure for infants with acute or chronic liver disease that is advanced, life-threatening, and unable to be adequately treated with other treatments such as drugs and surgery. The current meta-analysis was conducted to document patient survival, graft survival, as well as complications in infants undergoing liver transplantation. The findings of the meta-analysis indicate an overall 1-year patient survival rate of 85% and >5 year survival of 80%. For the graft survival, the 1 and >5 year rates were close to 70%. These pooled estimates seem satisfactory. We further observed that the patient and graft survival rates have improved considerably over time.

One of the intents of the review was also to understand whether there were temporal differences in the outcomes of interest, and the corresponding findings were interesting. The patient (87 vs. 81%) as well as graft survival (74 vs. 66%) rates at 1-year post-transplantation were higher in recently published studies compared to those published before the year 2000. With respect to the rates of complications, the most reported complication was infections (50%) followed by need for re-transplantation (16%) and biliary complications (15%). A comparatively higher complication rate was noted in studies done before the year 2000, especially for re-transplantation, biliary complications, and infection/sepsis. This seems to indicate that the recent advancements have largely taken care of the biliary tract complications and need for re-transplantation; however, the incidence of vascular complications is more or less the same, i.e., 12–14%.

The key message emerging from the meta-analysis is that while the short-term (1-year post-transplant) patient and graft survival is improving with time and is currently at an acceptable mark, focus should be placed on improving the survival rates in the long-term follow-up along with efforts to minimize the complication rates. Adequate mechanisms and management techniques should be instituted to lower down the incidence of infections and sepsis in post-transplant period. While it is exciting to note a moderate to high patient and graft survival rates following transplantation, it is also important to consider the long-term negative effects of immunosuppression. Studies have shown an increased risk of hypertension and nephrotoxicity in children with use of immunosuppressants such as cyclosporine, steroids, and tacrolimus (34–36). There also have been reports of failure in growth following steroid therapy in children (35, 37). These considerations are important while planning long-term rehabilitation of infants undergoing LT. Future studies should also take into account the quality of life in infants following liver transplantation. Currently, no data are available on this issue.

We also acknowledge that there could be problems with generalizing the findings of the overall pooled analysis as the number of critical factors could influence the outcomes of transplantation in infants. We therefore conducted a subgroup analysis, and we found that both patient survival and graft survival were better when the indication for transplant was biliary atresia/cholestasis and when the graft was received from a living donor. Furthermore, the outcomes were better when reduced/split graft was used, the weight of the child was ≥6 kg, and the age was ≥6 months at the time of transplant. These findings are important as they suggest that the success of transplantation varies based on these factors, and due consideration should be given to these at the time of planning for transplantation. It is possible that some centers do not perform transplantation in infants weighing under 5 kg or very early in infancy and therefore have good success rates. It is important to consider the patient and donor details carefully before evaluating the success or failure of transplantation.

One of the limitations of the review is the high degree of heterogeneity noted for most of the outcomes. The subgroup analysis indicated that majority of the heterogeneity was contributed by the pooling of studies done recently, i.e., those that were published since the year 2000. This implies requirement for more harmonized protocols for conducting follow-up studies on recipients of liver transplantation during infancy. Another limitation is that most of the included studies had a limited sample size. Studies with a small sample size are often met with the limitation of lack of generalizability of the findings. Consequently, there is a need for large studies with longer follow-up on diverse outcomes. Also, while we conducted a subgroup analysis based on a number of important factors such as the type of graft (e.g., whole or reduced/split; live/cadaveric donor; indication for transplant; age and weight of the recipient at the time of transplant), it is important to note that the included studies had not provided specific data pertaining to these identified subgroups. Rather the findings were provided for the study population as a whole. We therefore had to consider subgrouping studies based on the predominance of the categorizing factor. For instance, if in a study >50% of the infants underwent transplantation due to biliary atresia (and a smaller proportion for other indications), we considered pooling the findings of the study under the “indication for transplant as biliary atresia.” This approach may not give true pooled estimates but surely provides some indicative findings that might be useful.

The current meta-analysis showed that the outcomes in terms of patient and graft survival rates for infant liver transplantation are fairly good; however, there is scope for further improvement. The complication rates, especially for infection/sepsis post-transplant, are high. More studies with larger sample sizes and a longer duration of follow-up with considerations for a diverse range of outcomes are needed for comprehensive understanding.

## Data Availability Statement

Publicly available datasets were analyzed in this study. This data can be found here: PubMed, Scopus, and Google scholar databases.

## Author Contributions

YH and XW conceived and designed the study, and wrote the paper. HY and SZ were involved in literature search, data collection, analyzed the data, and reviewed and edited the manuscript. All authors read and approved the final manuscript.

## Conflict of Interest

The authors declare that the research was conducted in the absence of any commercial or financial relationships that could be construed as a potential conflict of interest.

## Supplementary Material

Supplementary Figure 1 Funnel plot for publication bias with respect to patient survival.

Supplementary Figure 2 Funnel plot for publication bias with respect to graft survival.

Supplemental Table 1 Search strategy for identification of studies to be included in the review.

Supplementary Table 2 Author's judgements about study quality using the adapted Ottawa-Newcastle Risk of Bias Assessment tool.

Supplementary Table 3 Key details of the studies included in the meta-analysis.
