# Supplementary figures and images for: Survival and Complication of Liver Transplantation in Infants: A Systematic Review and Meta-Analysis
Source: Front Pediatr. 2021 Apr 29;9:628771. doi: 10.3389/fped.2021.628771 (PMC8116516; doi:10.3389/fped.2021.628771)

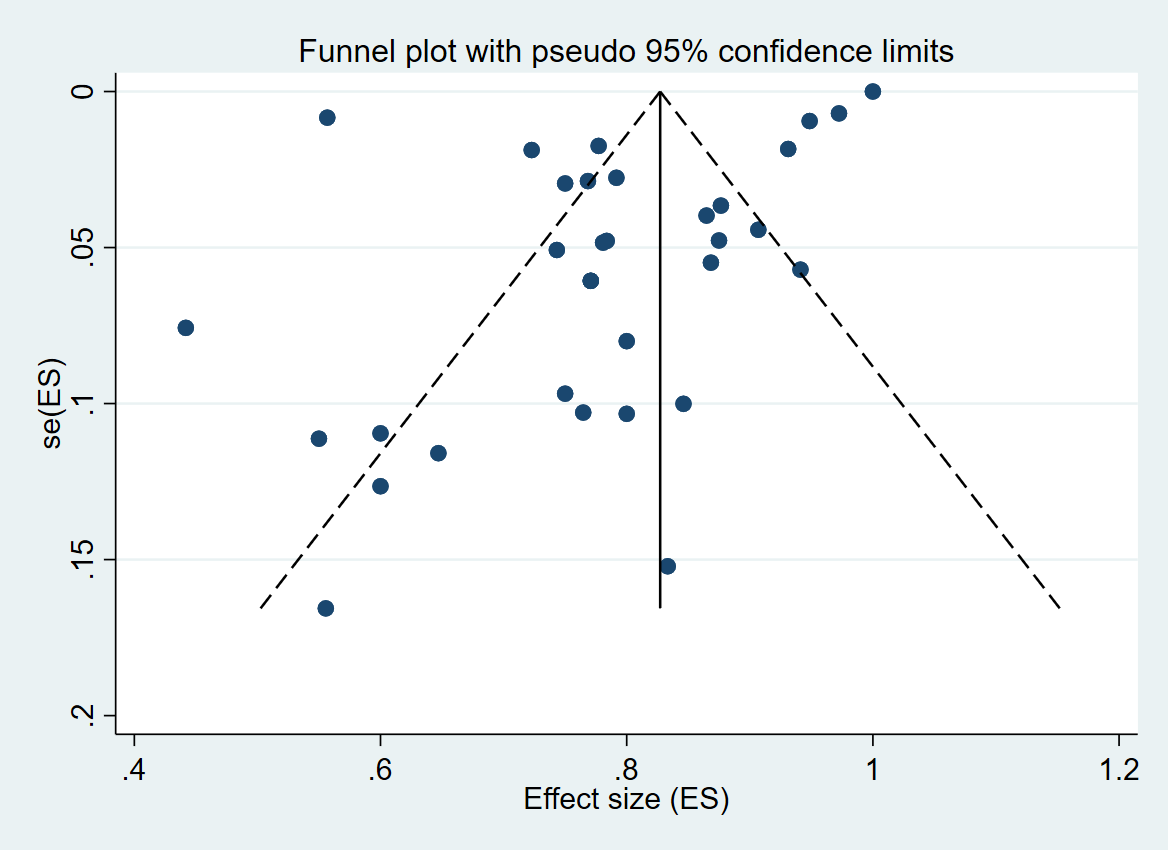

Supplement: Supplementary Figure 1 — Funnel plot for publication bias with respect to patient survival. [file Image_1.TIF]

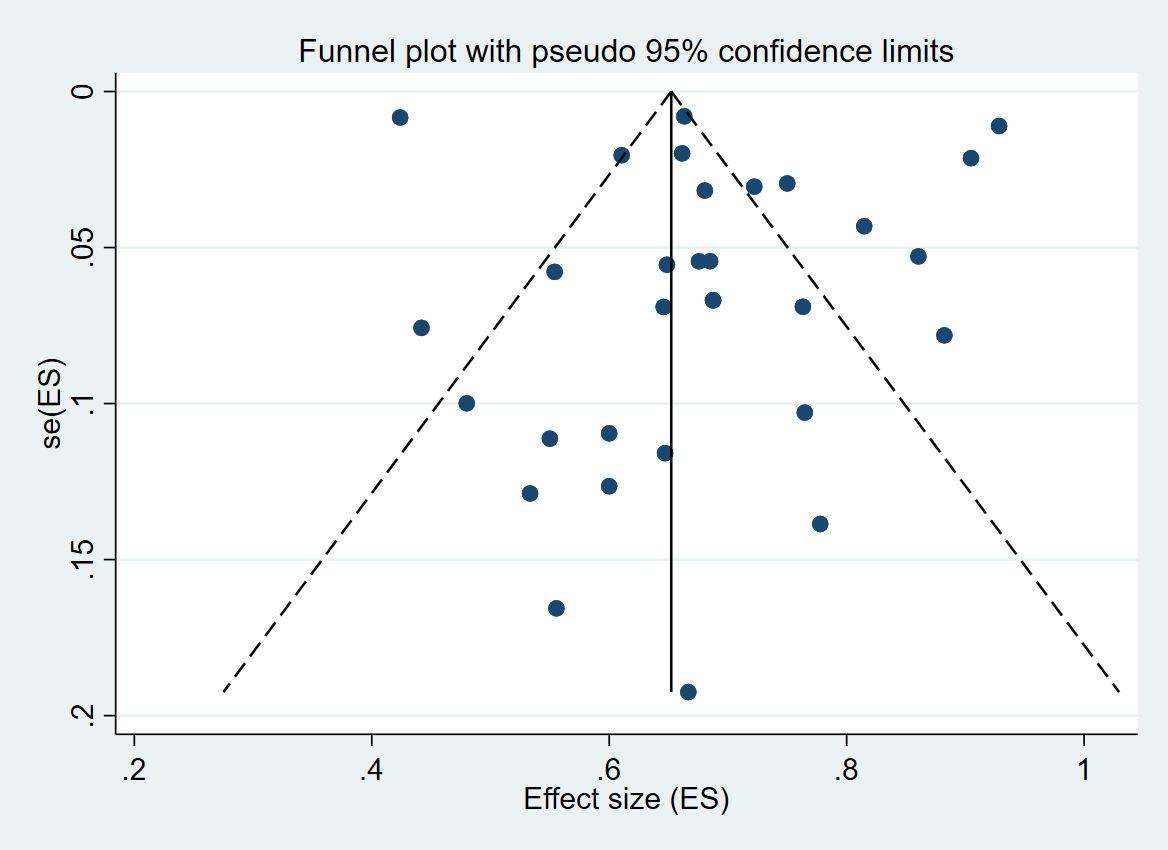

Supplement: Supplementary Figure 2 — Funnel plot for publication bias with respect to graft survival. [file Image_2.TIF]
