# Supplementary material for: Survival and Complication of Liver Transplantation in Infants: A Systematic Review and Meta-Analysis
Source: Front Pediatr. 2021 Apr 29;9:628771. doi: 10.3389/fped.2021.628771 (PMC8116516; doi:10.3389/fped.2021.628771)
Supplement: Supplemental Table 1 — Search strategy for identification of studies to be included in the review. [file Table_1.DOCX]

**Supplemental table 1. Search strategy for identification of studies to be included in the review**

| **Search strategy**  #1 (liver transplant OR hepatic transplant)  #2 (infants OR infancy OR neonatal OR newborn OR young children)  # 3 (survival OR patient survival OR graft survival OR complications OR adverse events)  #4 (Randomized controlled trial OR Controlled clinical trial OR prospective OR follow up OR retrospective OR observational OR quasi-experimental OR quasi-randomized)  #5 (#1 AND #2 AND #3 AND #4)  #6 (Addresses[ptyp] OR Autobiography[ptyp] OR Bibliography[ptyp] OR Biography[ptyp] OR pubmed books[filter] OR Case Reports[ptyp] OR Congresses[ptyp] OR Consensus Development Conference[ptyp] OR Directory[ptyp] OR Duplicate Publication[ptyp] OR Editorial[ptyp] OR Systematic reviews OR Meta analysis OR Festschrift[ptyp] OR Guideline[ptyp] OR In Vitro[ptyp] OR Interview[ptyp] OR Lectures [ptyp] OR Legal Cases[ptyp] OR News[ptyp] OR Newspaper Article[ptyp] OR Personal Narratives [ptyp] OR Portraits[ptyp] OR Retracted Publication[ ptyp] OR Twin Study[ptyp] OR Video-Audio Media[ptyp])  #7 (#5 NOT #6) |
| --- |
