# Supplementary material for: Survival and Complication of Liver Transplantation in Infants: A Systematic Review and Meta-Analysis
Source: Front Pediatr. 2021 Apr 29;9:628771. doi: 10.3389/fped.2021.628771 (PMC8116516; doi:10.3389/fped.2021.628771)
Supplement: Supplementary Table 2 — Author's judgements about study quality using the adapted Ottawa-Newcastle Risk of Bias Assessment tool. [file Table_2.DOCX]

**Supplementary table 2. Author’s judgements about study quality using the adapted Ottawa-Newcastle Risk of Bias Assessment tool**

|  | Venick RS et al | Tiao GM et al | Noujaim HM et al | Esquivel et al | Jain AK et al | Sokal EM et al | Sundaram SS et al | Grabhorn E et al | Kasahara et al | Jimenez-Rivera C et al | D’Alessandro AM et al |
| --- | --- | --- | --- | --- | --- | --- | --- | --- | --- | --- | --- |
| Representativeness/appropriateness of participant selection  Random or consecutive recruitment=Y  Convenience sample=N  Not reported or unclear | Y | Y | Y | Y | Y | N | Y | Y | Y | Y | Y |
| Control for baseline differences in cohorts  Similarity of groups at baseline or adjustment in analyses=Y  No attempt to control or adjust=N  Not reported=NR | Y | Y | N | N | Y | Y | Y | Y | Y | Y | Y |
| Loss to follow-up  Explanation provided for loss of participants and/or intention to treat=Y  No explanation =N | Y | N | Y | Y | N | Y | Y | Y | Y | Y | N |
| Masking of exposure to outcomes assessor  Description of masking=Y  No masking or no description =N | Y | Y | Y | Y | Y | Y | Y | Y | N | Y | Y |
| Ascertainment of condition  Description of ascertainment/diagnostic criteria=Y  No description or patient self-report=N | Y | Y | Y | Y | Y | N | Y | Y | Y | Y | Y |
| Documentation of other treatment modalities  Documentation=Y  No documentation=N | Y | Y | Y | N | Y | Y | Y | N | Y | Y | Y |
| Extent to which valid outcomes are described  Adequate description of outcome=Y  Insufficient detail regarding outcome or follow-up time=N | Y | Y | Y | Y | Y | Y | Y | N | Y | Y | Y |
| Prespecification of harms, mode of harms collection  Description of a list of harms assessed or monitoring=Y  No such description or passive harms collection=N  No adverse events reported=NA | Y | Y | Y | Y | Y | Y | Y | Y | Y | Y | Y |
| Financial Conflict of interest (COI)  Funding source reported=Y  Funding source not reported=N | N | N | Y | Y | Y | N | Y | N | Y | N | Y |

**Supplementary table 2 continued. Author’s judgements about study quality using the adapted Ottawa-Newcastle Risk of Bias Assessment tool**

|  | Arnon R et al | Mekeel KL et al | Lucianetti A et al | Dunn SP et al | Beath S et al | Colombani PM et al | Cacciarelli TV et al | Srinivasan P et al | Bonatti H et al | Van der Werf WJ et al | Saing H et al |
| --- | --- | --- | --- | --- | --- | --- | --- | --- | --- | --- | --- |
| Representativeness/appropriateness of participant selection  Random or consecutive recruitment=Y  Convenience sample=N  Not reported or unclear | Y | Y | Y | Y | Y | N | Y | Y | Y | Y | N |
| Control for baseline differences in cohorts  Similarity of groups at baseline or adjustment in analyses=Y  No attempt to control or adjust=N  Not reported=NR | Y | Y | N | N | Y | Y | Y | N | Y | Y | Y |
| Loss to follow-up  Explanation provided for loss of participants and/or intention to treat=Y  No explanation =N | Y | N | Y | Y | N | Y | N | Y | Y | Y | Y |
| Masking of exposure to outcomes assessor  Description of masking=Y  No masking or no description =N | Y | Y | Y | N | Y | Y | Y | Y | N | Y | N |
| Ascertainment of condition  Description of ascertainment/diagnostic criteria=Y  No description or patient self-report=N | Y | Y | N | Y | Y | N | Y | Y | Y | N | Y |
| Documentation of other treatment modalities  Documentation=Y  No documentation=N | Y | Y | Y | N | Y | Y | Y | N | Y | Y | Y |
| Extent to which valid outcomes are described  Adequate description of outcome=Y  Insufficient detail regarding outcome or follow-up time=N | Y | N | Y | Y | Y | N | Y | Y | Y | Y | Y |
| Prespecification of harms, mode of harms collection  Description of a list of harms assessed or monitoring=Y  No such description or passive harms collection=N  No adverse events reported=NA | Y | Y | Y | Y | Y | Y | Y | Y | Y | Y | Y |
| Financial Conflict of interest (COI)  Funding source reported=Y  Funding source not reported=N | N | N | Y | N | Y | Y | Y | N | N | N | Y |
