# Supplementary material for: Survival and Complication of Liver Transplantation in Infants: A Systematic Review and Meta-Analysis
Source: Front Pediatr. 2021 Apr 29;9:628771. doi: 10.3389/fped.2021.628771 (PMC8116516; doi:10.3389/fped.2021.628771)
Supplement: Supplementary Table 3 — Key details of the studies included in the meta-analysis. [file Table_3.docx]

**Supplementary table 3. Key details of the studies included in the meta-analysis**

| **Author, year of publication** | **Country** | **Data on Subjects** | **Sample size and study design** | **Key outcome** |
| --- | --- | --- | --- | --- |
| Venick RS et al (2010) (12) | USA | Infants with a mean age of 9 months at time of transplantation; mean (SD) weight of 7.2 (3.2) kg;  Reason for transplantation: majority with cholestatic jaundice (due to biliary atresia) (60%); majority received cadaveric graft (86%); Majority received whole graft (56%) | 216  Retrospective single centre cohort study | **Key findings**  *Graft survival rate*  1 yr: (162/216); 75%  5yr: (156/216); 72%  10 yr: (147/216); 68%  *Patient survival rate*  1 yr: (171/216); 79%  5 yr: (166/216); 77%  10 yr: (162/216); 75%  *Complications*  Hepatic artery thrombosis= (14/216); 6.5%  Portal vein thrombosis= (9/216); 4.2%  Requiring re-transplantation=(16/216); 7.4% |
| Tiao GM et al (2005) (13) | USA | Infants younger than one year (mean of 7.5 months); mean (range) weight of 6.4 (2.4 to 12.7) kg;  Reason for transplantation: majority with cholestatic jaundice (due to biliary atresia) (66.6%); majority received cadaveric graft (85%); Majority received reduced/split graft (44%) | 81  Retrospective review of the medical records | **Key findings**  *Graft survival rate*  1 yr: (66/81); 81%  *Patient survival rate*  1 yr: (71/81); 88%  *Complications*  Hepatic artery thrombosis= (5/81), 6.2%  Portal vein thrombosis= (8/81), 9.9%  Requirement for re-transplantation= (4/81), 4.9%  Infection= (11/81), 13.5% |
| Noujaim HM et al (2002) (14) | United Kingdom | Infants younger than one year (median age of 3 months) and weighed 5 kilograms or lower; Reason for transplantation: majority with acute hepatic failure/acute deterioration of chronic liver disease (87.0%); All received cadaveric graft; All received reduced/split graft | 15  Retrospective review of the records | **Key findings** (Median follow up of 34 months)  *Graft survival rate:* (8/15); 54%  *Patient survival rate:* (9/15); 60%  Complications  Hepatic artery thrombosis= (1/15), 6.7%  Portal vein thrombosis= (1/15), 6.7%  Requirement for re-transplantation= (1/15), 6.7%  Biliary complications=(2/15); 13.3%  Infections=(5/15); 33.3% |
| Esquivel et al (1987) (15) | USA | Infants under one year (age range of 3-11 months); mean weight of 7 kg; Reason for transplantation: biliary atresia (60.0%); All received cadaveric graft; All received whole graft | 20  Prospective follow up | **Key findings**  *Graft survival rate (~12 months):* (11/20); 55%  *Patient survival rate (~12 months):* (12/20); 60%  *5 yr patient survival rate:* (11/20); 55%  *Complications*  Hepatic artery thrombosis= (5/20), 25.0%  Biliary tract complications= (5/20), 25.0%  Requirement for re-transplantation= (6/20), 30.0%  Infection=(13/20); 65.0% |
| Jain AK et al (2020) (16) | USA | Infants under 12 months of age (mean age of 7.6 months); Majority had weight between 5-10 kg (81%); Majority received cadaveric graft (74%); reason for transplantation- cholestatic/biliary atresia (70%); Majority received reduced/split graft (60%) | 545  Data utilized from SPLIT registry that collected data prospectively | **Key findings**  *Patient survival rate*  *At 12 months:* (530/545); *97.3%*  *At 60 months:* (517/545); *94.8%*  *Graft survival rate at 60 months:* (506/545); *92.8%*  *Complications*  Hepatic artery thrombosis= (48/545), 8.8%  Portal vein thrombosis= (36/545), 6.6%  Biliary complications=(106/545); 19.5% |
| Sokal EM et al (1990) (17) | Belgium | Infants under 12 months of age; Mean age of 10.3 months and mean weight of 7.3 kg; Reason: Biliary atresia (88%); live donor; majority received reduced/split graft | 17  Prospective follow up of infants receiving liver transplant | **Key findings**  *Graft survival rate*  1 yr: (7/17); 41.2%  *Patient survival rate*  1 yr: (11/17); 64.7%  *Complications*  Hepatic artery thrombosis=(4/17);23.5 %  Requiring re-transplantation=(5/17); 29%  Infections=(15/17); 88.2% |
| Sundaram SS et al (2008) (18) | USA | Infants aged 0-90 days; mean weight of 3.9 kg; reason: fulminant hepatic failure or metabolic disease (74%); majority received from cadaveric donors (89.5%) and reduced/split graft | 38  Data queried from the Study of  Pediatric Liver Transplantation (SPLIT) database | **Key findings**  *Graft survival rate*  1 yr: (29/38); 76.3%  *Patient survival rate*  1 yr: (33/38); 86.8%  *Complications*  Hepatic artery thrombosis=(2/38);5.3 %  Portal vein thrombosis= (4/38); 10.5%  Infection=(20/38); 52.6%  Requiring re-transplantation=(23/38); 60.5%  Biliary tract complications=(8/38); 21.1% |
| Grabhorn E et al (2004) (19) | Germany | Infants aged 6 months or less; Mean age of 4.5 months and mean weight of 5.8 kg; Reason: Biliary atresia (62.8%); Majority with cadaveric donor (60%); majority received reduced/split graft | 43  Retrospective review of the records | **Key findings**  *Graft survival rate*  1 yr: (37/43); 86.0%  5 yr: (19/43); 44.2%  *Patient survival rate*  1 yr: (39/43); 90.7%  5 yr: (19/43); 44.2%  *Complications*  Hepatic artery thrombosis= (1/43); 2.33%  Portal vein thrombosis= (1/43); 2.33%  Requiring re-transplantation=(6/43); 14.0%  Infection=(33/43); 76.7%  Biliary tract complications=(6/43); 14.0% |
| Kasahara et al (2017) (20) | Japan | Infants aged under 12 months; median weight (6.75 kg); Reason- majority with cholestasis (70.0%) and received reduced graft; All received from live donor | 189  Prospective follow up of infants receiving liver transplant | **Key findings**  *Graft survival rate*  10 yr: (171/189); 90.4%  *Patient survival rate*  10 yr: (176/189); 93.1%  *Complications*  Portal vein thrombosis= (11/189); 5.8%  Biliary complications=(10/189); 5.3%  Infection=(41/189); 21.7% |
| Jimenez-Rivera C et al (2016) (21) | Canada | Infants aged 0-12 months; mean age of recipient 232 days (7.7 months); mean weight of 8.5 kg; reason: biliary atresia (65%); mostly received from cadaver and majority received whole graft (60%) | 3522  Retrospective cohort study conducted using data  from the UNOS database | **Key findings**  *Graft survival rate*  1 yr: (2336/3522); 66.3%  5 yr: (1493/3522); 42.4%  *Patient survival rate*  5 yr: (1961/3522); 55.7%  *Complications*  Re-transplantation=(538/3522); 15.2% |
| D’Alessandro AM et al (2007) (22) | USA | Infants aged 0-12 months; mean age of recipient 7.1 months; mean weight of 6.3 kg; reason: biliary atresia (85%); All received from cadaver and majority received whole graft (50%) | 74  Retrospective review of the medical records | **Key findings**  *Graft survival rate*  1 yr: (50/74); 67.6%  5 yr: (48/74); 64.8%  10 yr: (41/74); 55.4%  *Patient survival rate*  1 yr: (64/74); 86.4%  5 yr: (58/74); 78.3%  10 yr: (55/74); 74.3%  *Complications*  Hepatic artery thrombosis= (14/74); 18.9%  Portal vein thrombosis= (6/74); 8.1%  Biliary complications=(9/74); 12.1%  Infection=(28/74); 37.8%  Requiring re-transplantation=(14/74); 18.9% |
| Arnon R et al (2011) (23) | USA | Infants aged 0-12 months; mean age of recipient 1.3 months; mean weight of 4.32 kg; reason: biliary atresia (41.2%); All received from cadaver and majority received whole graft (57%) | 570  Retrospective study using data  from the UNOS database | **Key findings**  *Graft survival rate*  1 yr: (377/570); 66.1%  5 yr: (348/570); 61.1%  *Patient survival rate*  1 yr: (443/570); 77.7%  5 yr: (412/570); 72.2%  *Complications*  Hepatic artery thrombosis= (49/570); 8.6%  Portal vein thrombosis= (6/74); 8.1%  Biliary complications=(11/570); 1.9%  Infection=(148/570); 25.9%  Requiring re-transplantation=(70/570); 12.3% |
| Mekeel KL et al (2007) (24) | USA | Infants weighing 5 kg or less; mean age of recipient 4.4 months; reason: biliary atresia (45.0%); All received from cadaver and majority received reduced/split graft (61%) | 20  Retrospective review of the medical records | **Key findings**  *Graft survival rate*  5 yr: (12/20); 60.0%  *Patient survival rate*  5 yr: (15/20); 75.0%  *Complications*  Portal vein thrombosis= (3/20); 15.0%  Infection=(13/20); 65.0%  Requiring re-transplantation=(3/20); 15.0% |
| Lucianetti A et al (2005) (25) | Italy | Infants under 6.0 kg weight with a median age of 3 months; mean weight of 4.7kg; reason: majority with biliary atresia (53%); majority received split grafts (88%); all had cadaveric donors | 17  Prospective follow up of infants receiving liver transplant | **Key findings**  *Graft survival rate*  Within 1 yr: (15/17); 88.2%  At 6 years: (13/17): 77.0%  *Patient survival rate:*  Within 1 yr:(16/17); 94.0%  At 6 years: (13/17); 77.0%  *Complications*  Portal vein thrombosis= (1/17); 5.9%  Biliary complications=(5/17); 29.4% |
| Dunn SP et al (1993) (26) | USA | Infants under 1 year of age; mean age of 7.9 months; mean weight of 8.2kg; majority with biliary atresia (80%); Majority received whole graft from cadaveric donors | 15  Retrospective review of the medical records | **Key findings**  *Patient survival rate:*  At 1 year: (12/15); 80.0%  *Graft survival rate*  At 1 year: (9/15); 60.0%  *Complications*  Hepatic artery thrombosis= (1/15); 6.6%  Re-transplantation rate= (5/15); 33.3% |
| Beath S et al (1994) (27) | UK | Infants under 1 year of age; mean age of 9 months; mean weight of 7.0 kg; majority with biliary atresia (76%); Majority received reduced graft (90%) and from cadaveric donors | 25  Prospective follow up of infants receiving liver transplant | **Key findings** (follow up till 4 years)  *Patient survival rate:* (20/25); 80.0%  *Graft survival rate*: (12/25); 48.0%  *Complications*  Hepatic artery thrombosis= (2/25); 8.0%  Biliary complications=(7/25); 28.0%  Infection and sepsis=(18/25); 72.0%  Re-transplantation rate= (3/25); 12.0% |
| Colombani PM et al (1996) (28) | USA | Infants with age ranging between 4-11 months; mean age of 7.5 months; mean weight of 6.6 kg; majority with biliary atresia (85%); Majority received reduced graft (85%) and from living donors (>50%) | 13  Retrospective review of medical records | **Key findings** (mean follow up of around 20 months)  *Patient survival rate:* (11/13); 85.0%  *Complications*  Re-transplantation rate= (3/13); 23.1%  Infections: (10/13);77.0%  Hepatic artery thrombosis= (3/13); 23.1%  Biliary complications=(3/13); 23.1% |
| Cacciarelli TV et al (1997) (29) | USA | Infants under 1 year of age; majority weighing >=4 kg (93%); majority in the age group of 5-11 months; majority with biliary atresia (74%); Majority received reduced graft (60%) and from cadaveric donors | 73  Retrospective data from records (?) | **Key findings**  *Patient survival rate at 1 yr:* (57/73); 78.0%  *Graft survival rate at 1 yr*: (50/73); 68.0%  Hepatic artery thrombosis= (9/73); 14.0 %  Portal vein thrombosis=(1/73); 1.34% |
| Srinivasan P et al (1999) (30) | UK | Infants with median age of 37.5 days; median weight of 3.45 kg; majority with neonatal hemochromatosis (67%); Majority received reduced/split graft from cadaveric donors | 6  Prospective follow up of infants receiving liver transplant | **Key findings** (mean follow up of around 30 months)  *Patient survival rate:* (5/6); 83.3%  *Graft survival rate*: (4/6); 66.7%  Complications  Hepatic artery thrombosis: (1/6); 16.7%  Re-transplantation rate: (1/6); 16.7%  Infection: (2/6); 33.3% |
| Bonatti H et al (1997) (31) | UK | Infants under 3 months of age; mean age of 6 weeks; mean weight of 3.7 kg; majority with fulminant hepatic failure due to hemochromatosis/hepatitis (67%); All received reduced graft from live donors | 9  Prospective (?) follow up of infants receiving liver transplant | **Key findings** (mean follow up of around 22 months)  *Patient survival rate:* (5/9); 55.5%  *Graft survival rate*: (5/9); 55.5%  Complications  Infection: (5/9); 55.5%  Biliary complications: (1/9); 11.1%  Hepatic artery thrombosis: (1/9); 11.1%  Portal vein thrombosis: (1/9): 11.1% |
| Van der Werf WJ et al (1998) (32) | USA | Infants under 12 months of age; mean weight of 6.2 kg; majority with biliary atresia (>50%); Majority received whole graft (>50%) and from cadaver | 48  Retrospective review of the medical records | **Key findings**  *Graft survival rate*  1 yr: (33/48); 68.7%  5yr: (33/48); 68.7%  10 yr: (31/48); 64.5%  *Patient survival rate*  1 yr: (42/48); 87.5%  5 yr: (37/48); 77.1%  10 yr: (37/48); 77.1%  Complications  Re-transplantation: (13/48); 27.1%  Biliary complications: (5/48); 10.4%  Hepatic artery thrombosis: (9/48); 18.7%  Portal vein thrombosis: (5/48): 10.4%  Infection: (6/48); 12.5% |
| Saing H et al (1999) (33) | Hong Kong | Infants under 12 months of age; mean age of 9.5 months; mean weight of 7.03 kg; majority with biliary atresia (89%); Majority received segmental graft from live donors (67%) | 9  Retrospective review of the medical records | **Key findings** (mean follow up of 40 months)  *Graft survival rate:* (7/9); 87.5%  *Patient survival rate*: (9/9); 100.0%  Complications  Re-transplantation: (1/9); 11.1%  Biliary complications: (3/9); 33.3%  Infection: (3/9); 33.3% |
